# Genotype-driven identification of a molecular network predictive of advanced coronary calcium in ClinSeq® and Framingham Heart Study cohorts

**DOI:** 10.1186/s12918-017-0474-5

**Published:** 2017-10-26

**Authors:** Cihan Oguz, Shurjo K. Sen, Adam R. Davis, Yi-Ping Fu, Christopher J. O’Donnell, Gary H. Gibbons

**Affiliations:** 1 Cardiovascular Disease Section, National Human Genome Research Institute, National Institutes of Health, Bethesda, MD USA; 2 Office of Biostatistics Research, Division of Cardiovascular Sciences, National Heart, Lung and Blood Institute, National Institutes of Health, Bethesda, MD USA; 3 Framingham Heart Study, Boston University School of Medicine, Boston, MA USA; 4 Center for Population Genomics, MAVERIC, VA Healthcare System, Boston, MA USA; 5 Cardiology Section Administration, VA Healthcare System, Boston, MA USA; 6 Department of Cardiology, Massachusetts General Hospital, Harvard Medical School, Boston, MA USA; 7 Office of the Director, National Heart, Lung and Blood Institute, National Institutes of Health, Bethesda, MD USA

**Keywords:** Coronary artery calcium, Random forest, Neural networks, Case-control study, Coronary heart disease, Genotype data, Systems biology

## Abstract

**Background:**

One goal of personalized medicine is leveraging the emerging tools of data science to guide medical decision-making. Achieving this using disparate data sources is most daunting for polygenic traits. To this end, we employed random forests (RFs) and neural networks (NNs) for predictive modeling of coronary artery calcium (CAC), which is an intermediate endo-phenotype of coronary artery disease (CAD).

**Methods:**

Model inputs were derived from advanced cases in the ClinSeq®; discovery cohort (n=16) and the FHS replication cohort (n=36) from 89^*th*^-99^*th*^ CAC score percentile range, and age-matched controls (ClinSeq®; n=16, FHS n=36) with no detectable CAC (all subjects were Caucasian males). These inputs included clinical variables and genotypes of 56 single nucleotide polymorphisms (SNPs) ranked highest in terms of their nominal correlation with the advanced CAC state in the discovery cohort. Predictive performance was assessed by computing the areas under receiver operating characteristic curves (ROC-AUC).

**Results:**

RF models trained and tested with clinical variables generated ROC-AUC values of 0.69 and 0.61 in the discovery and replication cohorts, respectively. In contrast, in both cohorts, the set of SNPs derived from the discovery cohort were highly predictive (ROC-AUC ≥0.85) with no significant change in predictive performance upon integration of clinical and genotype variables. Using the 21 SNPs that produced optimal predictive performance in both cohorts, we developed NN models trained with ClinSeq®; data and tested with FHS data and obtained high predictive accuracy (ROC-AUC=0.80-0.85) with several topologies. Several CAD and “vascular aging" related biological processes were enriched in the network of genes constructed from the predictive SNPs.

**Conclusions:**

We identified a molecular network predictive of advanced coronary calcium using genotype data from ClinSeq®; and FHS cohorts. Our results illustrate that machine learning tools, which utilize complex interactions between disease predictors intrinsic to the pathogenesis of polygenic disorders, hold promise for deriving predictive disease models and networks.

**Electronic supplementary material:**

The online version of this article (doi:10.1186/s12918-017-0474-5) contains supplementary material, which is available to authorized users.

## Background

Informed medical decision making through the effective use of clinical and genomic data is one of the promising elements of personalized precision medicine [1] in which predictive models enable the assessment of alternative treatment strategies [2]. Predictive models also play a pivotal role in utilizing the genomic data for generating predictions regarding the disease risk and progression [3–5] with the potential to generate biological insights into the mechanisms behind complex diseases [6], such as coronary artery disease (CAD). In CAD, the arteries of the heart, which supply oxygen rich blood to the cardiac muscle, lose their ability to function properly due to atherosclerosis. CAD is a multifactorial disease [7, 8] that has been associated with many clinical and demographic variables, and major risk factors such as high blood pressure, high levels of blood lipids, smoking and diabetes. Our study focuses on coronary artery calcium (CAC), which is an intermediate endo-phenotype of CAD [9]. The level of CAC, which is measured by the CAC score, varies within a broad range in the general population. CAC score is a strong predictor of lethal cardiac events, including myocardial infarction (MI) [10–15]. A major objective of personalized precision medicine is to identify subgroups of patients that are at the highest risk of cardiovascular events and accelerated vascular aging, such as patients with highly advanced CAC, among a large population of patients at intermediate risk based on standard clinical variables.

The key mechanism behind coronary artery calcification is the phenotypic modulation of vascular cells that is triggered by stimuli including oxidative stress, increased rate of cell death [16], and high levels of inflammatory mediators [17]. The genetics behind CAC deposition is complex. Several important genes involved in vascular calcification have been previously identified through mouse model studies [18], studies on rare human diseases that lead to excessive calcification [17], and through elucidation of its links with bone mineralization [19]. Several genome-wide association studies (GWAS) have also previously focused on CAC [20–25]. Some of the human genomic loci linked to CAC are *9p21*, *PHACTR*, and *PCSK9* (also linked to CAD and MI [22, 26, 27]). Several past studies have combined clinical variables and genotype data for predicting CAD. Some examples include implementation of Cox regression models [28–30] and the use of allele counting, logistic regression, and support vector machines in [31]. Statistical modeling of CAC as an intermediate phenotype for CAD has also been the subject of research in recent years [32, 33].

Recently, there has been increasing interest in the application of machine learning methods for predicting disease subphenotypes by utilizing genomic features [34]. These methods provide increased ability for integrating disparate sources of data while utilizing interactions (both linear and nonlinear) between genomic features (e.g., gene-gene interactions) [35]. Machine learning methods eliminate the need for multiple testing correction required in statistical association tests that treat each predictor separately. They also mitigate potential biases that could originate from model misspecification since machine learning typically aims at identifying model structures that are optimal for the training data [36].

In this study, we utilized machine learning tools for predictive modeling of the advanced CAC subphenotype by integrating clinical variables and genotype data. Our study focused on identifying predictors of the high-risk subgroup of CAD patients with advanced CAC among an intermediate risk sample of middle-aged Caucasian males. Previous studies have established that higher CAC scores are observed among men compared to women [37, 38], as well as a higher prevalence of CAC among white Americans compared to black Americans [39].

We used the random forest (RF) algorithm, which is a decision tree based machine learning method [40] established as an effective tool for modeling with genomic data [41] to develop predictive models for the subset of individuals with advanced CAC. We derived model inputs (or SNPs) using two feature selection approaches. First, we leveraged a literature based strategy based on previous association studies of CAC to define a set of 57 single nucleotide polymorphisms (SNPs). As an alternative contextual approach, we utilized a standard feature selection and filtering approach in machine learning to identify 56 additional SNPs from the ClinSeq®; genotype data [42, 43]. We assessed the predictive performances of these sets of SNPs with and without clinical variables in the ClinSeq®; cohort. For validation of the observed predictive patterns, we evaluated these SNP sets in an independent sample set from the Framingham Heart Study (FHS) and identified a robust subset of predictive SNPs that performed consistently well in data sets from both cohorts. Using this subset of SNPs, we developed neural network (NN) models trained with data from the ClinSeq®; discovery cohort and tested with data from the FHS replication cohort under a wide range of network topologies, and assessed the predictive performances of these models. The biological processes enriched in the molecular network of genes constructed from the predictive loci generated insights into potential mediators of advanced CAC, which is a distinct subphenotype of vascular disease.

## Methods

### Overview of the computational analysis

**Fig. 1:**
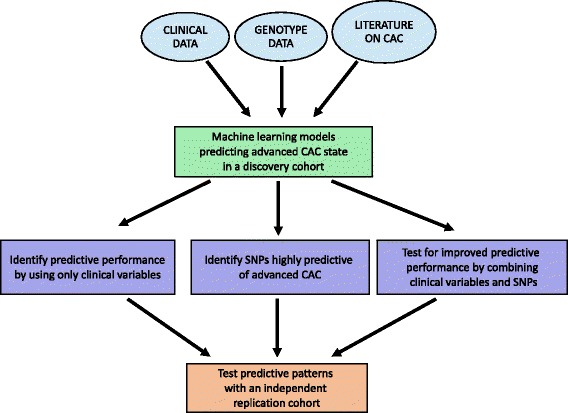
Overall strategy of the analysis

Our overall strategy was to use clinical data and genotype data for predicting advanced CAC in a discovery cohort, and to test if the observed predictive patterns can be confirmed in an independent cohort (Fig. 1). We developed RF models that predict advanced CAC within the ClinSeq®; cohort using traditional risk factors (or clinical variables) and then derived two sets of SNPs. The first one was a set of GWAS-identified SNPs (or “SNP Set-1”) previously associated with CAC, whereas the second set (or “SNP Set-2”) was derived using genotype data from the ClinSeq®; discovery cohort. In order to limit the number of SNPs in SNP Set-2, we used a standard feature selection approach in machine learning [44, 45] and extracted the 56 SNPs (among 668,427 SNPs) whose genotypes had the highest Pearson correlation values with the advanced CAC phenotype. We assessed the predictive performance by using only clinical data (to establish a baseline performance) and only genotype data, as well as their combination.

After assessing the RF based predictive patterns generated by the clinical variables, SNP Set-1 and SNP Set-2 in the ClinSeq®; discovery cohort, we focused on testing the most predictive set of SNPs in the FHS replication sample. Based on the analysis of predictive performance and replication in both sample sets, we identified the subset of SNPs that generated optimal performance in RF models in both cohorts. As an additional validation of the robustness of our findings, we trained and tested NN models with the genotypes of these SNPs in the ClinSeq®; and FHS cohorts, respectively. Data used in NN models came from advanced CAC cases and age-matched controls (all Caucasian males) in both cohorts.

**Fig. 2:**
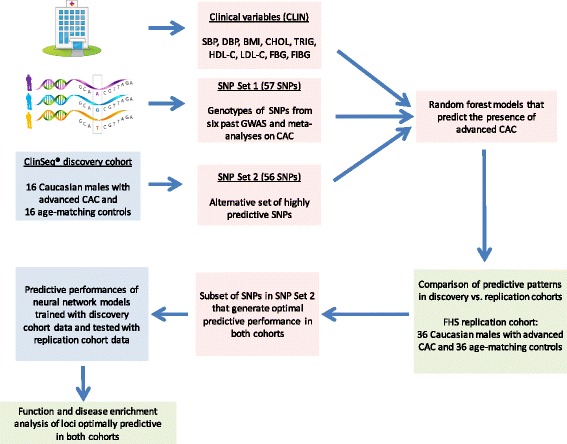
Schematic of the modeling approach

Upon verifying the high predictive performance under a wide range of NN topologies, we utilized GeneMANIA [46] to create a functional interaction network composed of genes on which this subset of SNPs were located, as well as additional genes known to be most closely related to these genes. GeneMANIA uses linear regression to maximize the connectivity between the genes within the network while minimizing the interactions with the genes that are excluded. Two types of links between gene pairs were found to be present in this network: co-expression (correlated expression levels) and genetic interactions (effects of a gene perturbation can be changed by a second perturbed gene). Gene Expression Omnibus (GEO) and Biological General Repository for Interaction Datasets (BioGRID) are the main sources of co-expression and genetic interaction datasets, respectively in the GeneMANIA database. Finally, using the list of genes within this network derived by GeneMANIA, we performed function and disease enrichment analysis to demonstrate the relevance of this molecular network to cardiovascular disease based on existing knowledge in the literature. Figure 2 illustrates the steps taken in our analysis.

### CAC scores and binary CAC states

The models we developed in this study aimed at predicting the binary case-control statuses of age-matched Caucasian male patients. Hence, we first transformed the CAC scores (measured by Agatston method [47]) of the 32 Caucasian male subjects from the ClinSeq®; study that formed our discovery cohort (data previously published in [42, 43]) into binary CAC states. 16 control subjects in this cohort had zero CAC scores corresponding to state “0", whereas the 16 age-matched cases had high CAC scores (ranging between 500 and 4400) corresponding to state “1". These binary case-control states served as the true class labels and were later used for training and testing of the developed classification models. Based on the Multi-Ethnic Study of Atherosclerosis (MESA) cohort standards [48, 49], a percentile value for each case was computed using the online MESA calculator that takes age, gender, race and CAC score as its inputs. The case subjects in the ClinSeq®; discovery cohort, two of which were diabetic, fell within the 89^*th*^-99^*th*^ CAC score percentile range.

The replication cohort from FHS comprised of 36 controls and 36 age-matched Caucasian male case subjects (including three diabetic cases) also within the 89^*th*^-99^*th*^ CAC score percentile range. As an additional set of comparative control groups, 122 cases from FHS within 29^*th*^-88^*th*^ CAC score range were split into two distinct sets of 61 cases within 29^*th*^-68^*th*^ and 69^*th*^-88^*th*^ percentile ranges and were age-matched with two sets of 61 subjects with no CAC. These two equal-sized subcohorts were then used to test whether the predictive patterns generated by the discovery (ClinSeq®;) and replication (FHS) cohorts were specific to the 89^*th*^-99^*th*^ percentile CAC score range and not replicable with lower levels of coronary calcium. Two classes of model variables were used in this study as predictors of coronary calcium, namely clinical variables and genotypic variables, as described below.

### Clinical variables

Nine clinical variables available from all subjects in both cohorts were utilized as predictors of CAC. These variables included body mass index (BMI), cholesterol levels (low-density lipoprotein (LDL), high-density lipoprotein (HDL), and total cholesterol), triglycerides, blood pressure (systolic and diastolic), fasting blood glucose level, and fibrinogen. All subjects were non-smoker Caucasian males in both ClinSeq®; and FHS cohorts. The detailed description of each clinical variable is given in Additional file 1: Table S1, whereas the mean and standard deviation values among cases vs. controls, along with their *p*-values are listed in Additional file 1: Tables S2 and S3 for ClinSeq®; and FHS cohorts, respectively.

### Genotypic variables

We compiled two sets of SNPs using a feature selection strategy that relied on the existing CAC literature, as well as the ClinSeq®; discovery cohort. The first set of 57 SNPs were reported in previous association studies of CAC that focused on the presence of CAC rather than its extreme levels [20–25]. We named this set “SNP Set-1” (listed in Additional file 1: Table S4 along with the reported *p*-values). From the the ClinSeq®; genotype data, we also generated a second set of 56 SNPs (“SNP Set-2”) as described above. All SNPs in SNP Set-2 are listed in Additional file 1: Table S5. Genotypes of the 113 biallelic SNPs in both SNP sets were coded as 0 or 2 (homozygous for either allele) or 1 (heterozygous) using the same reference alleles in both ClinSeq®; and FHS cohorts. Details regarding the genotyping protocols and data sources for both cohorts are provided in Additional file 2: Supplementary Text.

### Predictive modeling using RFs and NNs

We implemented the RF classification method using the Statistics and Machine Learning Toolbox ^TM^ of Matlab^®;^ [50] for predicting the binary CAC state. Predictive accuracy is computed by generating receiver operating characteristic (ROC) curves (true positive rate vs. the false positive rate obtained using several classifier output thresholds) and by quantifying the areas under these curves (AUC). Due to the randomized nature of the classification method, we performed 100 runs (per set of features or model inputs) and reported the mean AUC (normality of the AUC distributions not rejected by Anderson-Darling tests [51]). For each reported AUC value, we empirically derived a *p*-value as the fraction of AUC values in 1000 runs (with randomly permuted case-control statuses) at or above the mean AUC value generated when the case-control statuses are not permuted (i.e., the actual data). This approach has been previously used for computing the statistical significance of ROC-AUC values [32, 52]. For machine learning based classification models with two classes (e.g., cases and controls), the baseline predictive performance from ROC curves is AUC=0.5 (commonly used AUC threshold in clinical studies that look at sensitivity and specificity of classifiers [53]) corresponding to a classification likelihood of a coin flip.

For each decision tree, approximately two-thirds of the data (this ratio varied up to ±15% among different runs) is retained to be used for model training, whereas the remaining data is used for model testing. These test samples are referred to as “out-of-bag” (OOB) samples, whereas the training samples are expanded by bootstrapping [54] (or sampling with replacement) up to the sample size of the original data [55] prior to model training. Classification of the test samples are based on the complete ensemble of trees (a total of 100 trees) with the “majority vote” scheme [56]. For example, a test sample is predicted to be “CAC positive” if the number of trees that predict “State 1” is higher than the ones that predict “State 0”. Predictive importance is computed for each input variable by permuting its values corresponding to the test subjects and finding the change in the prediction error (or the fraction of incorrectly classified subjects). In mathematical terms, the prediction error for OOB samples without permutation (*e*_*OOB*_) is computed as *n*_*m,OOB*_/(*n*_*c,OOB*_+*n*_*m,OOB*_), where *n*_*m,OOB*_ and *n*_*c,OOB*_ stand for the numbers of misclassified and correctly classified samples without permutation, respectively. Likewise, the prediction error for OOB samples with permuted input values (*e*_*OOB,perm*_) is computed as *n*_*m,OOB,perm*_/(*n*_*c,OOB,perm*_+*n*_*m,OOB,perm*_), where *n*_*m,OOB,perm*_ and *n*_*c,OOB,perm*_ stand for the numbers of misclassified and correctly classified samples with permutation, respectively. The difference between the two error terms (*e*_*OOB,perm*_−*e*_*OOB*_) is computed for each tree and the average value of this difference (over all trees) is divided by its standard deviation to identify the predictive importance of a feature. Features with positive predictive importance have higher *e*_*OOB,perm*_ values in comparison with their *e*_*OOB*_ values.

Features are ranked with respect to their cumulative predictive importance evaluated from 100 independent runs, or RF models. Stronger predictors have higher predictive importance values than weaker predictors. After ranking all features in each distinct feature set (e.g., all clinical variables), we decreased the number of features gradually by leaving out weaker predictors to identify the optimal predictive performance and the corresponding optimal set of features. We repeated this procedure to compare the predictive performances of models trained and tested by combining clinical and genotype data, as well as using each layer data in isolation. The predictive patterns generated by data from the ClinSeq®; discovery cohort were also compared with the patterns generated by the independent FHS replication cohort. Finally, RF models were also used to identify a subset of SNPs in SNP Set-2 that generated the optimal predictive performance in both ClinSeq®; and FHS cohorts.

Upon identifying the subset of SNPs in SNP Set-2 that generate RF models with optimal performance in both cohorts, we further validated our results by implementing a neural network (NN) based classification approach using the NN Toolbox^TM^ of Matlab^®;^ [50]. This allowed us to test whether the cumulative predictive signal captured by RFs is also captured by a different method that does not rely on decision trees and to assess the robustness of the predictive signal in our data set. In addition, NN implementation allowed us to test several network topologies while using discovery/replication cohort samples for training/testing these topologies (rather than using the randomized OOB sampling of RFs). Further details regarding the rationale behind our RF-NN implementation are provided in Additional file 2: Supplementary Text.

We trained three-layer feedforward networks using backpropagation [57] with sigmoid transfer functions in two hidden layers and a linear transfer function in the output layer. In both hidden layers, the number of nodes was varied from one to 20 with increments of one, thereby leading to a total of 400 network configurations individually used for training and testing. In short, the inputs into each network layer (initial input is the genotype data) are weighted and the sum of the weighted inputs transformed by the transfer functions of the hidden layers are used to generate model outputs (or the case/control status) [58]. We trained all network configurations with the genotypes of the optimal subset of SNPs within SNP Set-2 from the advanced CAC cases and age-matched controls in the ClinSeq®; discovery cohort. Approximately 20% of the training samples include the “validation” samples used for minimizing overfitting during training. We subsequently performed model testing with the genotype data from the advanced CAC cases and age-matched controls subjects in the FHS replication cohort.

Predictive accuracy was once again assessed with ROC curves. For each NN configuration, we computed the median AUC value (normality of the AUC distributions rejected by Anderson-Darling tests [51]) among 100 independent runs. Once again, we derived an empirical *p*-value based on the predictive performance obtained from 1000 runs with randomized case-control statuses.

## Results

### Models built with clinical variables and SNP Set-1

**Fig. 3:**
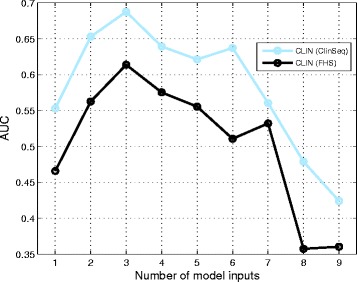
Predictive performance plotted against the number of predictors in ClinSeq®; and FHS cohorts. Model inputs are derived only from clinical variables

We first built RF models using all of the nine clinical variables from the ClinSeq discovery cohort and identified that three of them had positive predictive importance values as listed in Table 1. These predictors included HDL Cholesterol, systolic blood pressure, and fibrinogen. Fibrinogen has been previously associated with CAC [59, 60] as a critical biomarker of inflammation [61] and atherosclerosis [62]. Within the FHS replication cohort, five clinical variables including total cholesterol, systolic and diastolic blood pressure, fibrinogen and fasting blood glucose (a glycemic trait previously associated with CAC levels [63]) had positive predictive importance values. As we varied the number of predictors between one to nine, the optimal AUC values were 0.69 (*p*-value=0.015) and 0.61 (*p*-value=0.080) for ClinSeq®; and FHS cohorts, respectively (Fig. 3). These AUC values were within the range of 0.60-0.85, which is the previously reported AUC range compiled from 79 studies predicting CAD or cardiac events based on the Framingham risk score (FRS) [64]. Even though our case-control sample was already stratified by age and gender, the remaining clinical variables still exhibited modest predictive value.

**Table 1:** Predictive importance values of clinical variables in ClinSeq®; and FHS cohorts. Only the instances with positive predictive importance are reported

Clinical variable	Predictive importance
Total cholesterol	8.60 (FHS)
Systolic blood pressure	6.24 (FHS), 12.94 (ClinSeq®;)
Diastolic blood pressure	2.88 (FHS)
Fibrinogen	1.81 (FHS), 3.50 (ClinSeq®;)
Fasting Blood Glucose	0.024 (FHS)
HDL cholesterol	18.39 (ClinSeq®;)

We next built RF models for the ClinSeq®; discovery cohort using the literature-derived genotypes of the 57 SNPs in “SNP Set-1” as model inputs and identified 17 SNPs with positive predictive importance. To compare the predictive patterns generated by the discovery and replication cohorts based on the SNP Set-1 genotype data, we next developed RF models for the FHS replication cohort and identified 19 SNPs among SNP Set-1 with positive predictive importance in this cohort. Top 30 percentile predictors in SNP Set-1 (i.e., predictive SNPs) generated AUC ranges of 0.68-0.72 and 0.71-0.78 in ClinSeq®; and FHS cohorts (without clinical variables), respectively. Only five of the 17 predictive SNPs (29%) from the ClinSeq®; discovery cohort were predictive in the FHS cohort pointing to a low degree of replication between the two cohorts. In order the test whether the combination of the nine clinical variables and SNP Set-1 resulted in improved predictive performance, we merged these two groups of model inputs with the ClinSeq®; discovery data set. We observed a significant improvement in the AUC range from 0.68-0.72 (only SNP Set-1) to 0.72-0.77 (combined set of inputs). In contrast, when we used the FHS replication data set in the same way, AUC range declined from 0.71-0.78 to 0.69-0.75. Hence, the improvement of predictive accuracy we observed within the ClinSeq®; discovery cohort, by adding clinical variables to SNP Set-1, was not confirmed in the FHS replication cohort.

### Selection of SNP Set-2 based on genotype-phenotype correlation within the ClinSeq®; discovery cohort

Although the literature-based SNP Set-1 provided a useful initial source of model inputs, we recognized that a potential limitation of this approach was the focus of past association studies on CAC as a broad and heterogeneous phenotype. In contrast, our study aims to derive an optimal set of predictors for the subset of CAC positive patients with the most advanced vascular lesions at the top decile of the broad CAC score range. Accordingly, we employed a standard feature selection approach to derive an alternative set of genotypes (SNP Set-2) from the ClinSeq®; data that were highly correlated with the advanced CAC subphenotype (described in Methods). This approach effectively leverages the capacity of RF algorithm to eliminate non-informative signals and sort out input SNPs of potential predictive utility without the multiple-testing penalty. The range of genotype-phenotype correlation among the SNPs in SNP Set-2 (no overlap with SNP Set-1) was 0.63-0.73 within the ClinSeq®; discovery cohort. Upon incorporating the genotypes of SNP Set-2 in this cohort into RF models, we obtained an AUC value of 0.9975. Given this high predictive performance, our subsequent analyses focused on further validation and refinement of this set of genotypes.

### Predictive performance of SNP Set-2 in FHS and ClinSeq®; data sets

In order to test whether the high predictive performance of SNP Set-2 was replicated in the FHS cohort, we trained and tested RF models using the genotypes of SNP Set-2 in the replication cohort. We identified that the positive predictive importance values of 30 of the 56 predictive SNPs (54%) were replicated. We also observed common patterns between the discovery and replication cohorts in terms of the predictive importance based rankings of the 30 SNPs with positive predictive importance in both cohorts. Nine of the top 18 SNPs overlapped between the two cohorts, whereas the top two SNPs (rs243170 and rs243172, both on *FOXN3*) were the same in both cohorts.

Top 30 SNPs, which were selected based on their positive predictive importance in both cohorts, generated AUC ranges of 0.80-0.85 and 0.96-0.99 in the replication and discovery cohorts, respectively. Hence, SNP Set-2 was highly predictive in both discovery and replication cohorts. Combining the clinical variables and SNP Set-2 did not improve the predictive performance in either cohort. In fact, there was a slight decline in the optimal AUC from 0.85 to 0.83 in the FHS cohort, whereas no change in the optimal AUC was observed in the ClinSeq®; cohort with the combination of clinical variables and SNP Set-2 (Table 2).

**Table 2:** Predictive performances of RF models (quantified by the mean ± standard deviation values of AUC) trained and tested with different predictor sets in the ClinSeq®; and FHS cohort data

Predictors	Optimal # markers	Optimal AUC	*p*-value
CLIN	3 (ClinSeq®;), 3 (FHS)	0.69 ±0.02 (ClinSeq®;), 0.61 ±0.02 (FHS)	0.015 (ClinSeq®;), 0.080 (FHS)
SNP Set-2	21 (ClinSeq)®;, 21 (FHS)	0.99 ±0.01 (ClinSeq®;), 0.85 ±0.02 (FHS)	<0.001 (ClinSeq®;), <0.001 (FHS)
CLIN+SNP Set-2	21 (ClinSeq®;), 18 (FHS)	0.99 ±0.01 (ClinSeq®;), 0.83 ±0.01 (FHS)	<0.001 (ClinSeq®;), <0.001 (FHS)

“CLIN” corresponds to the nine clinical variables listed in Additional file 1: Table S1 (all variables except age and gender)

One potential explanation of the high predictive performance of SNP Set-2, which does not include any SNPs previously associated with CAC, in both cohorts is the broad range of CAC levels. Given that SNP Set-2 was derived from cases with extreme levels of CAC, it remained to be determined whether the predictive power of SNP Set-2 was specific to this extreme phenotype or whether it could be generalized to a broader range of CAC levels. Hence, we tested the collective predictive performance of the 30 SNPs in SNP Set-2 that had positive predictive power in both cohorts with genotype data from cases with lower levels of CAC. Among the 61 cases within the 29^*th*^-68^*th*^ percentile range and the 61 age-matched controls, top 50 percentile markers generated an AUC range of 0.62-0.66. Utilizing the data from 61 cases within 69^*th*^-88^*th*^ range and 61 age-matched controls, AUC range was approximately the same (0.61-0.66). These results further extended the robustness of our findings and demonstrated that the high predictive performance of SNP Set-2 was only observed in the 89^*th*^-99^*th*^ percentile CAC score range.

### Subset of SNPs in SNP Set-2 with optimal predictive performance in both cohorts and enrichment analysis

**Fig. 4:**
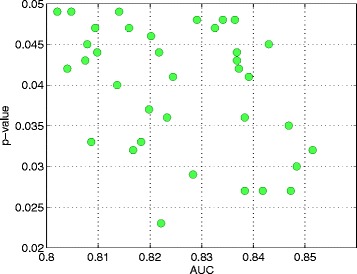
Properties of 36 optimal NN models trained with data from the discovery cohort and tested with data from the replication cohort. Median AUC value for each network topology (ranging between 0.8021 and 0.8515) and the corresponding *p*-values. Third quartile of the AUC values among different network topologies ranged between 0.8503 and 0.9074

Table 3 shows the list of 21 SNPs in SNP Set-2 generated optimal predictive performance in ClinSeq®; and FHS cohorts. Using the genotypes of these 21 SNPs, we trained NN models of 400 distinct topologies with ClinSeq®; data and tested each topology with the FHS data. As shown in Fig. 4, we obtained 36 model topologies with AUC values ranging between 0.80-0.85 with empirically derived *p*-values of less than 0.05, thereby utilizing a different machine learning approach to further validate the collective predictive ability of these SNPs in the FHS replication cohort. This result demonstrates the stable and consistent features of these 21 SNPs in predicting advanced CAC independent of the classifier strategy employed. The optimal NN topologies have 9-20 nodes in their first hidden layers and 6-20 nodes in their slightly less complex second hidden layers.

**Table 3:** Predictive importance values of the set of SNPs that generate optimal predictive performance in both cohorts. Nearest genes are listed for intergenic SNPs (marked with asterisk)

SNP	Locus	Predictive importance	Predictive importance	Percent
		(ClinSeq®;)	(FHS)	difference
rs13159307	*FBXL17* ^*^	28.83	21.64	24.94
rs8107904	*EMR2* ^*^	36.95	21.83	40.92
rs571797	*NRG3*	17.68	6.86	61.20
rs2390285	*MACC1*	22.86	17.27	24.45
rs342393	*NRG3*	18.04	15.34	14.97
rs13429160	*LOC101927701*	35.68	16.89	52.66
rs11674863	*LOC101927701*	26.18	15.74	39.88
rs514237	*NRG3*	19.09	24.81	23.06
rs6860493	*NNT*	20.72	26.39	21.49
rs10054519	*C5orf28*	21.17	25.25	16.16
rs12521249	*PAIP1* ^*^	21.17	25.44	16.78
rs10065689	*NNT*	20.45	25.55	19.96
rs2241097	*TLR5*	34.02	24.11	29.13
rs10059993	*NNT-AS1*	20.82	24.77	15.95
rs12645809	*ANTXR2*	22.1	25.33	12.75
rs480220	*NRG3*	19.76	24.01	17.70
rs1366410	*NNT*	21.15	23.77	11.02
rs11767632	*YAE1D1* ^*^	32.09	20.94	34.75
rs7713479	*NNT-AS1*	21.11	37.48	43.68
rs243172	*FOXN3*	34.90	46.17	24.41
rs243170	*FOXN3*	35.91	51.20	29.86

The normalized difference of the predictive importance values of each SNP in two cohorts (difference divided by the higher predictive importance value in the two cohorts) has a median value of 24% (interquartile range:17%-36%). In terms of predictive importance based ranking, five of the top 11 SNP predictors (with 65% of the cumulative predictive importance) are common, whereas nine of the top 14 SNP predictors (with 76% of the cumulative predictive importance) overlap between two cohorts

^*^Intergenic SNPs for which the nearest genes are reported

**Fig. 5:**
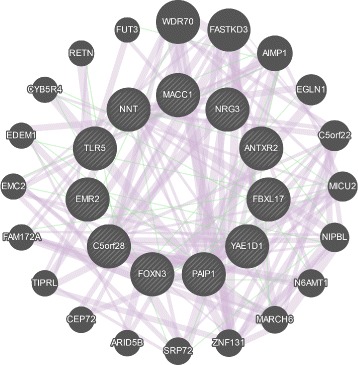
Network of genes derived from GeneMANIA (based on 244 studies in humans) using the most predictive set of SNPs in this study. The connections in pink are derived from gene coexpression data, whereas the connections in green are derived from genetic interaction data from the literature. The inner circle is composed of genes on which the subset of SNPs in SNP Set-2 leading to optimal performance in both cohorts are present, whereas the genes forming the outer circle are additional genes identified by GeneMANIA. The thicknesses of the links (or edges) between the genes are proportional to the interaction strengths, whereas the node size for each gene is proportional to the rank of the gene based on its importance (or gene score) within the network. All interactions within this network are listed in Additional file 1: Table S8

We identified a total of 13 genes that included the 21 SNPs leading to optimal predictive performance in both cohorts. Using GeneMANIA, we derived a molecular network that included this group of 13 genes in addition to the 18 genes known to be linked to the first group based on coexpression and genetic interaction data from the literature [46]. Figure 5 shows this network, whereas the abbreviated gene symbols and the corresponding gene names are listed in Additional file 1: Table S6. The proteins coded by the genes in the network have a wide range of roles. Twelve of them are either a transcription factor or an enzyme, one is a translational regulator, and two are transmembrane receptors.

In order to identify whether gene list was enriched in any biological functions or processes associated with CAD, we used two bioinformatics resources, namely Database for Annotation, Visualization and Integrated Discovery (DAVID) [65] and Ingenuity Pathway Analysis (IPA, Qiagen, Redwood City, CA, USA). Through their associations with blood magnesium levels, type-2 tumor necrosis factor receptors, HDL cholesterol, BMI, CAD, and adiponectin, 17 of the 31 genes in our network are associated with only one disease class, namely cardiovascular disease with a 1.9 fold-enrichment and a *p*-value of 0.0025 (modified Fisher’s exact test) based on DAVID and the Genetic Association Database. Furthermore, through mouse and rat models, six genes in our network (*ARID5B*, *CYB5R4*, *EGLN1*, *RETN*, *TLR5*, and *NRG3*) have been previously associated with several CAC risk factors including diabetes, insulin resistance, LDL cholesterol, and triglycerides (all associations listed in Additional file 2: Supplementary Text). Table 4 and Additional file 1: Table S7 show the cardiovascular disease related biological functions and phenotypes (identified by IPA based on Fisher’s exact test with *p*-value <0.05), that are enriched within our network. Several biological processes enriched among the network genes are associated with “vascular aging” (further discussion in the next section).

**Table 4:** Enriched diseases and biological functions (in the network of genes derived from GeneMANIA) with *p*-values ranging between 1.0E-4 and 1.0E-2 as identified by IPA based on Fisher’s exact test

Category	Disease or function	Genes	*p*-value
Connective tissue development and function	Quantity of adipose tissue	*ARID5B, CYB5R4*	3.58E-4
		*RETN, TLR5*	
Connective tissue development and function	Differentiation of adipocytes	*ARID5B, EGLN1*	8.82E-4
		*NIPBL, RETN*	
Cardiovascular disease	Angiectasis of blood vessel	*EGLN1*	9.87E-4
Cardiovascular system development and function	Area of capillary vessel	*EGLN1*	9.87E-4
Hematological system development and function	Cell division of	*AIMP1*	9.87E-4
	peripheral blood lymphocytes		
Cardiovascular disease, endocrine system disorders,	Susceptibility to insulin	*RETN*	9.87E-4
Metabolic disease	resistance-related hypertension		
Cardiac necrosis, cell death and survival	Cell death of heart tissue	*EGLN1*	1.97E-3
Cellular movement	Migration of connective tissue cells	*AIMP1, ARID5B*	2.14E-3
		*RETN*	
Carbohydrate metabolism, cellular function	Homeostasis of D-glucose	*CYB5R4, RETN*	2.46E- 3
and maintenance		*TLR5*	
Nucleic acid metabolism	Conversion of NAD+	*NNT*	2.96E- 3
Cardiovascular system development and function	Tethering of endothelial cell lines	*FUT3*	2.96E-3
Cellular compromise, inflammatory response	Degranulation of beta islet cells	*CYB5R4*	3.94E-3
Cardiovascular system development and function	Density of blood vessel tissue	*AIMP1*	3.94E-3
Endocrine system disorders, hematological disease	Onset of hyperglycemia	*CYB5R4*	3.94E-3
Metabolic disease			
Carbohydrate metabolism	Tolerance of D-glucose	*CYB5R4*	4.93E- 3
Cardiovascular system development and function	Angiogenesis of heart	*EGLN1*	5.91E-3
Cardiovascular system development and function	Density of blood vessel	*AIMP1, EGLN1*	5.96E-3
Immune cell trafficking, inflammatory response	Adhesion of neutrophils	*ADGRE2* (*EMR2*)	7.52E-3
Hematological system development and function		*TLR5*	
Endocrine system development and function	Insulin sensitivity of liver	*RETN*	7.87E-3
Hepatic system development and function			
Nucleic acid metabolism	Metabolism of NADPH	*CYB5R4*	7.87E-3
Connective tissue development and function	Quantity of visceral fat	*RETN*	8.85E- 3
Carbohydrate metabolism	Binding of chondroitin sulfate	*ADGRE2* (*EMR2*)	9.83E-3

51 additional enriched diseases and biological functions (statistically less significant) with *p*-values ranging between 1.0E-2 and 5.0E-2 are listed in Additional file 1: Table S7

## Discussion

A major goal in the cardiovascular disease field is identifying individuals who are at greatest risk of accelerated CAD pathogenesis and complications, such as stroke and MI. Recognizing that the utility of traditional risk factors (particularly those driven by age) is not sufficiently robust to identify all patient groups with accelerated CAD [66], incorporating genomic data into machine learning tools for building predictive models of CAD is a promising area with potential clinical applications in future studies [2]. To this end, our study has demonstrated the utility of using a machine learning approach to identify a panel of SNPs predictive of a complex polygenic trait observed among a high-risk subset of patients. The resulting set of SNPs generated higher performance over traditional risk factors in predicting advanced CAC in a replicable manner in two independent cohorts.

In a previous study [33], authors combined clinical variables with 13 predictive SNPs from 13 different genes (identified among 2882 candidate SNPs from 231 genes that were proposed by a group of MESA investigators) for predicting the presence of coronary calcium using a Bayesian approach. None of these 13 SNPs were included in SNP Set-1 since they were not associated with CAC in a past GWAS or meta-analysis. Likewise, SNP Set-2 did not include any of these SNPs since their genotypes in the ClinSeq discovery cohort were not correlated highly enough with the binary advanced CAC state to pass our feature selection filter. A key difference between our study and [33] is the severity of the CAC scores among case subjects. The cases in [33] had CAC scores around 50th percentile (based on the reported average age and CAC score), whereas CAC scores of our cases fell within the top decile CAC score range defined by the MESA cohort data [48, 49]. While SNP Set-2 (derived from our discovery cohort) was highly predictive of advanced CAC in the FHS replication cohort, its predictive power declined significantly with cases that had lower CAC levels in the same cohort.

Understanding the drivers of accelerated CAD pathogenesis hold great potential for providing insights into inflammatory and immune responses [67–69] beyond conventional mediators (e.g., dysregulation of lipid metabolism and blood pressure) [67, 70]. Excessive reactive oxygen species (ROS) generation has been previously linked to high CAC levels [71, 72] and vascular aging [73]. Through ROS activity, macrophages that contain lipid molecules (or foam cells) accumulate in the artery walls and promote atherosclerosis [74]. *EMR2* is a network gene that promotes the release of inflammatory cytokines from macrophages and has been reported to be highly expressed in foamy macrophages handling lipid overload in atherosclerotic vessels [75]. Excessive ROS generation also leads to reduced bioactivity of nitric oxide (NO) [76], which is a cardioprotective molecule. The reduced form of NADP (NADPH) is required for the synthesis of cholesterol [77] as a cofactor in all reduction reactions. It is also required for the regeneration of reduced glutathione (GSH) [78] that provides protection against ROS activity [79]. Two of our network genes, *NNT* (associated with diabetes in mice [80]) and *CYB5R4*, are both involved in NADPH metabolism. As key elements of NADPH metabolism, NADPH oxidases generate ROS and are considered as therapeutic targets against vascular aging [81]. NADPH oxidase activity has been shown to modulate atherosclerosis in mice [82].

Among our network genes previously associated with arterial aging, *TLR5* is a member of the TLR (toll-like receptor) family, which is an established mediator of atherosclerosis [83] due to its role in immune response through the induction of inflammatory cytokines [84]. *RETN* is a biomarker for metabolic syndrome. Its overexpression has been shown to lead to increased atherosclerotic progression in mice [85]. Similarly, inhibition of *EGLN1* has been shown to provide protection against atherosclerosis in mice by improving glucose and lipid metabolism and reducing inflammation and decreasing the areas of atherosclerotic plaque [86]. HIF1-alpha proteins, which are modulated by *EGLN1*, are established regulators of inflammation and atherosclerosis [87].

*NRG3* is a network gene that is a member of the neuregulin family. Another member of this family is *NRG1*, which has been shown to inhibit atherogenesis and macrophage foam cell formation in a human study [88]. It has also been shown to moderate the association between job strain and atherosclerosis among men [89]. Another network gene *FOXN3* has been associated with fasting blood glucose, serum cholesterol, and diabetes in past GWAS [90–92]. *FOXN3* has also been linked to carotid intima-media thickness (a subclinical measure for atherosclerosis) and plaque in recent fine mapping studies in humans [93, 94]. Taken together, our findings show that several biological processes and risk factors associated with cardiovascular disease, and particularly with vascular aging, are enriched within the network we derived from the loci of SNPs that are highly predictive of advanced CAC. Vascular aging is highly relevant to CAC since aged vascular smooth muscle cells (VSMCs) are known to have less resistance against phenotypic modulations that promote vascular calcification [95]. In fact, along with seven traditional risk factors (age, gender, total cholesterol, HDL cholesterol, systolic BP, smoking status, hypertension medication status), the Agatston CAC score is used as a parameter in quantifying “vascular age” in the MESA arterial age calculator [96].

Dividing case subjects into subcategories based on the level of disease measured by different measures such as CAC scores, to pursue subphenotype-specific models [67] is a potentially effective approach for studying heart disease phenotypes. In this predictive modeling study, we focused on case subjects within the 89^*th*^-99^*th*^ percentile CAC score range and age-matched controls in two patient cohorts. The replication of highly predictive loci identified from the ClinSeq discovery cohort in the FHS cohort and the fact that we observe enrichment of several biological processes previously linked to cardiovascular disease at the network level demonstrates the effectiveness of our machine learning based approach. Our analysis provides a candidate list for conventional genotype-phenotype association studies of advanced CAC without the genome wide multiple testing penalty, thereby illustrating the complementary utility of machine learning and regression-based methods that can provide inputs to each other for follow-up studies.

## Conclusions

We used a combination of clinical and genotype data for predictive modeling of advanced coronary calcium. Machine learning models trained with SNP Set-2 (identified from the ClinSeq discovery cohort) produced high predictive performance in the FHS replication cohort. Upon identifying a subset of 21 SNPs from this set that led to optimal predictive performance in both cohorts, we developed NN models trained with the ClinSeq genotype data. We tested these models with the FHS genotype data and obtained high predictive accuracy values (AUC=0.80-0.85) under a wide range of network topologies, thereby replicating the collective predictive ability of these SNPs in FHS. At the gene network level, several biological processes previously linked to cardiovascular disease, including processes associated with accelerated “vascular aging”, were found to be enriched among the predictive loci.

A potential extension of our modeling study is the expansion of the panel of SNPs, which are highly predictive of advanced CAC levels, around their loci for building more comprehensive models. Subsequently, we would like to test these potential predictors of rapid CAC progression and early onset of MI with longitudinal data in independent cohorts, especially for cases poorly predicted by traditional risk factors. To conclude, our study on CAC, a cardiovascular disease phenotype and a predictive marker of future cardiac events illustrates the potential of combining multiple machine learning methods as informative and accurate diagnostic tools. Our results also suggest that utilizing markers specific to a limited range of coronary calcium, rather than its complete spectrum, is an effective approach for building accurate predictive models for personalized medicine efforts that require disease-level specific risk prediction and prevention.

## Additional files

Additional file 1 Supplementary Tables. This pdf file includes supplementary tables referred to in the main text. (PDF 184 kb)

Additional file 2 This pdf file provides information about the genotype data from ClinSeq®; and FHS cohorts, rationale behind random forest and neural network implementation for modeling advanced CAC, and mouse and rat model-based associations between the predictive network genes and cardiovascular disease processes and risk factors. (PDF 165 kb)

## Abbreviations

AUC: Area under the curve
BMI: Body mass index
BioGRID: Biological general repository for interaction datasets
CAC: Coronary artery calcium
CAD: Coronary artery disease
CHARGE: Cohorts for heart and aging research in genomic epidemiology
DAVID: Database for annotation, visualization and integrated discovery
ECM: Extracellular matrix
FHS: Framingham heart study
FRS: Framingham risk score
GSH: Reduced glutathione
GWAS: Genome-wide association studies
GEO: Gene expression omnibus
HWE: Hardy-Weinberg equilibrium
HDL: High-density lipoprotein
IPA: Ingenuity pathway analysis LDL: Low-density lipoprotein
MESA: Multi-ethnic study of atherosclerosis
MI: Myocardial infarction
NN: Neural network
NO: Nitric oxide
OOB: Out-of-bag
RF: Random forest
ROC: Receiver operating characteristics
ROC-AUC: Area under receiver operating characteristic curve
ROS: Reactive oxygen species
SHARe: SNP Health Association Resource
TLR: Toll-like receptor
VSMCs: Vascular smooth muscle cells
WGA: Whole genome amplification
