# Supplementary material for: Genotype-driven identification of a molecular network predictive of advanced coronary calcium in ClinSeq® and Framingham Heart Study cohorts
Source: BMC Syst Biol. 2017 Oct 26;11:99. doi: 10.1186/s12918-017-0474-5 (PMC5659034; doi:10.1186/s12918-017-0474-5)
Supplement: Supplementary file 1 — Supplementary Tables. This pdf file includes supplementary tables referred to in the main text. (PDF 184 kb) [file 12918_2017_474_MOESM1_ESM.pdf]

Table S1: **Clinical variables**

Number	Variable	Description
1	Age	-
2	Gender	-
3	Systolic BP (mmHg)	Maximum arterial pressure during contraction of the left ventricle of the heart.
4	Diastolic BP (mmHg)	Minimum arterial pressure when the ventricles of the heart fill with blood.
5	BMI (kg/m^2)	A person's weight in kilograms divided by the square of height in meters.
6	Fasting Blood Glucose (mg/dL)	Level of glucose in the blood after an overnight fast.
7	Total Cholesterol (mg/dL)	Calculated as LDL+HDL+Triglycerides/5, used to assess heart disease risk.
8	Triglycerides (mg/dL)	Lipids that are used to store energy and give energy to muscles.
9	HDL Cholesterol	Moves excess cholesterol from the cells to the liver.
10	LDL Cholesterol	Transports lipids around the body within the water outside cells.
11	Fibrinogen	A coagulation factor that is essential for blood clot formation.

Except age and gender, all clinical variables are used for predicting coronary calcium within random forest models in conjunction with SNP predictors.

Table S2: **General statistics of clinical variables among controls and case subjects (89th-99th CAC score percentile) in the ClinSeq® cohort**

Number	Variable	p-value	Cases mean	Cases SD	Controls mean	Controls SD
1	Age	0.82	58.25	4.70	57.88	4.33
2	Gender	-	All males	-	All males	-
3	Systolic BP (mmHg)	0.51	129.91	10.45	126.97	14.18
4	Diastolic BP (mmHg)	0.70	78.06	7.04	76.91	9.33
5	BMI (kg/m^2)	0.09	28.74	5.03	26.17	2.79
6	Fasting Blood Glucose (mg/dL)	0.07	106.44	16.74	97.69	8.34
7	Total Cholesterol (mg/dL)	0.78	190.75	49.02	186.75	30.88
8	Triglycerides (mg/dL)	0.17	127.13	62.69	98.75	49.77
9	HDL Cholesterol (mg/dL)	0.11	49.38	11.59	56.00	11.44
10	LDL Cholesterol (mg/dL)	0.74	115.63	38.28	111.44	30.88
11	Fibrinogen (mg/dL)	0.37	312.38	33.28	299.63	45.61

Mean and standard deviation (SD) values of the clinical variables in the ClinSeq® cohort. Per variable, a p-value is computed using a two-sample t-test (with univariate criterion) by testing the null hypothesis that the case and control vectors are from populations with equal means without making the assumption that the two populations have equal variance values for any predictor.

Table S3: **General statistics of clinical variables among controls and case subjects (89th-99th CAC score percentile) in the FHS cohort**

Number	Variable	p-value	Cases mean	Cases SD	Controls mean	Controls SD
1	Age	0.58	51.28	5.57	50.53	5.98
2	Gender	-	All males	-	All males	-
3	Systolic BP (mmHg)	0.10	125.86	17.07	120.00	12.03
4	Diastolic BP (mmHg)	0.72	78.56	10.32	77.81	6.67
5	BMI (kg/m^2)	0.21	28.41	4.44	27.23	3.38
6	Fasting Blood Glucose (mg/dL)	0.07	102.25	10.78	98.06	8.39
7	Total Cholesterol (mg/dL)	0.97	194.22	36.50	193.89	31.34
8	Triglycerides (mg/dL)	0.65	130.33	72.43	122.89	64.47
9	HDL Cholesterol (mg/dL)	0.86	46.78	13.05	46.25	12.76
10	LDL Cholesterol (mg/dL)	0.81	121.38	32.00	123.06	28.43
11	Fibrinogen (mg/dL)	0.09	361.86	97.01	329.33	58.59

Mean and standard deviation (SD) values of the clinical variables in the FHS cohort. Per variable, a p-value is computed using a two-sample t-test (with univariate criterion) by testing the null hypothesis that the case and control vectors are from populations with equal means without making the assumption that the two populations have equal variance values for any predictor.

Table S4: **List of SNPs associated with CAC in past GWAS and meta-analyses (SNP Set 1)**

Number	SNP	Locus	p-value	GWAS	Number	SNP	Locus	p-value	GWAS
1	rs4456611	*BCL2*	4.1E-4	[1]	37	rs34014631	*C10orf76*	4.3E-6	[2]
2	rs3789422	*ABCA4*	8.5E-5	[1]	38	rs11777747	*FLJ43860*	7.9E-6	[2]
3	rs13386681	*ATOH8*	1.6E-4	[1]	39	rs9506514	*IFT88* *	4.9E-6	[2]
4	rs10499276	*OPRM1* *	6.9E-5	[1]	40	rs7070038	*IPMK* *	6.6E-6	[2]
5	rs13260	*COL4A1*	8.7E-5	[1]	41	rs7765175	*MARCKS* *	4.7E-6	[2]
6	rs6667260	*ITPKB*	8.0E-5	[1]	42	rs10502575	*MCART2* *	1.5E-7	[2]
7	rs3768991	*NPAS2*	3.7E-4	[1]	43	rs10803016	*PLD5*	7.4E-6	[2]
8	rs17056112	*ADRA1A*	3.3E-4	[1]	44	rs16976171	*RIT2* *	3.6E-6	[2]
9	rs4977574	*CDKN2B-AS1*	9.0E-5	[1]	45	rs7856675	*SLC1A1*	8.3E-6	[2]
10	rs2891168	*CDKN2B-AS1*	1.7E-4	[1]	46	rs9907236	*SOX9* *	1.7E-6	[2]
11	rs10757274	*CDKN2B-AS1*	2.2E-4	[1]	47	rs2622633	*ZFPM2*	6.5E-6	[2]
12	rs10757272	*CDKN2B-AS1*	3.5E-4	[1]	48	rs1537370	*CDKN2B-AS1*	2.3E-11	[3]
13	rs2834669	*RUNX1*	3.9E-4	[1]	49	rs9349379	*PHACTR1*	2.7E-11	[4]
14	rs10483853	*NUMB*	6.1E-6	[5]	50	rs163189	*PRDM6*	7.5E-3	[4]
15	rs10519394	*PCDH18* *	1.1E-5	[5]	51	rs599839	*PSRC1*	3.9E-3	[4]
16	rs9321354	*TAAR5* *	3.4E-5	[5]	52	rs646776	*CELSR2*	7.9E-12	[4]
17	rs220457	*C17orf79* *	3.8E-4	[5]	53	rs2259816	*HNF1A*	4.8E-7	[4]
18	rs722208	*ESR1*	5.0E-3	[5]	54	rs3184504	*SH2B3*	8.6E-8	[4]
19	rs1365057	*CD44* *	7.7E-3	[5]	55	rs9818870	*MRAS*	7.4E-13	[4]
20	rs1467558	*CD44*	2.0E-2	[5]	56	rs11206510	*PCSK9*	9.6E-9	[4]
21	rs2190305	*CREB5*	1.2E-6	[6]	57	rs12146493	*DKFZp761E198*	7.4E-3	[4]
22	rs9574536	*SPRY2* *	2.4E-6	[6]					
23	rs7158225	*FLRT2* *	5.3E-6	[6]					
24	rs17819063	*FTO*	4.2E-5	[6]					
25	rs432695	*CDYL* *	2.3E-7	[6]					
26	rs4867326	*CDH6* *	4.7E-7	[6]					
27	rs622348	*MIR759* *	3.8E-6	[6]					
28	rs16872734	*GPR125*	7.0E-6	[6]					
29	rs2727551	*PRKAG2*	5.2E-5	[6]					
30	rs10096362	*CDH17* *	2.2E-6	[6]					
31	rs9843942	*TGFBR2*	3.0E-6	[6]					
32	rs1679195	*LPP*	3.4E-5	[6]					
33	rs2618157	*THBS1* *	5.3E-5	[6]					
34	rs6032769	*SLC35C2* *	1.3E-5	[6]					
35	rs1062087	*TBC1D4*	4.2E-7	[6]					
36	rs3758014	*ARHGEF10*	7.8E-5	[6]					

Genotypes of these SNPs (previously associated with CAC) are used for predicting the binary CAC state among the ClinSeq® subjects. Nearest genes are listed for intergenic SNPs (marked with asterisk).

Table S5: **List of highly predictive SNPs not previously associated with coronary calcium in past GWAS and meta-analyses (SNP Set 2)**

Number	SNP	Locus	Number	SNP	Locus
1	rs243170	*FOXN3*	37	rs8107904	*EMR2**
2	rs243172	*FOXN3*	38	rs9290557	*NAALADL2*
3	rs328395	*LOC100506172**	39	rs9804449	*FGF3**
4	rs342393	*NRG3*	40	rs9967032	*DOK6*
5	rs480220	*NRG3*	41	rs10036954	*C5orf34*
6	rs514237	*NRG3*	42	rs10054519	*C5orf28*
7	rs571797	*NRG3*	43	rs10059993	*NNT-AS1*
8	rs750582	*C5orf28**	44	rs10065689	*NNT*
9	rs1130329	*RBAK-LOC389458*	45	rs10079672	*AFAP1L1*
10	rs1288331	*SLC1A7**	46	rs10458221	*EEF1E1-BLOC1S5**
11	rs1366410	*NNT*	47	rs11131194	*RPUSD3*
12	rs1389401	*ASB7**	48	rs11575624	*HABP2*
13	rs1505279	*C15orf54**	49	rs11575634	*HABP2*
14	rs1887413	*TTL**	50	rs11674863	*LOC101927701*
15	rs2217855	*FBXO8**	51	rs11767632	*YAE1D1**
16	rs2241097	*TLR5*	52	rs12521249	*PAIP1**
17	rs2390285	*MACC1*	53	rs12645809	*ANTXR2*
18	rs4255867	*DOK6*	54	rs13159307	*FBXL17**
19	rs4410460	*KCNH8**	55	rs13220973	*LOC401324**
20	rs4491835	*SCN11A**	56	rs13429160	*LOC101927701*
21	rs4622486	*C15orf54**			
22	rs4632970	*AGK*			
23	rs4676660	*WDR48**			
24	rs4793853	*MSI2*			
25	rs6014727	*CASS4*			
26	rs6024879	*CASS4*			
27	rs6069753	*CASS4*			
28	rs6451696	*C5orf28*			
29	rs6565484	*RPTOR*			
30	rs6849832	*DDIT4L**			
31	rs6860493	*NNT*			
32	rs7176702	*MEGF11*			
33	rs7225157	*DNAH9*			
34	rs7501899	*C17orf54**			
35	rs7531219	*PPP2R5A**			
36	rs7713479	*NNT-AS1*			

Nearest genes are listed for intergenic SNPs (marked with asterisk *).

Table S6: **The network of genes derived by GeneMANIA from a database of 244 studies in humans. The loci of the 21 predictive SNPs are used as the input gene set for network construction. Gene symbols and names are based on the information from the HUGO Gene Nomenclature Committee (HGNC).**

Number	Gene symbol	Gene name
1	*MACC1*	Metastasis Associated In Colon Cancer 1
2	*NRG3*	Neuregulin 3
3	*ANTXR2*	Anthrax Toxin Receptor 2
4	*FBXL17*	F-Box And Leucine-Rich Repeat Protein 17
5	*YAE1D1*	Yae1 Domain Containing 1
6	*PAIP1*	Poly(A) Binding Protein Interacting Protein 1
7	*FOXN3*	Forkhead Box N3
8	*C5orf28*	Chromosome 5 Open Reading Frame 28
9	*EMR2* (*ADGRE2*)	EGF-like module-containing mucin-like hormone receptor-like 2 (Adhesion G Protein-Coupled Receptor E2)
10	*TLR5*	Toll Like Receptor 5
11	*NNT*	Nicotinamide Nucleotide Transhydrogenase
12	*WDR70*	WD repeat domain 70
13	*FASTKD3*	FAST Kinase Domains 3
14	*AIMP1*	Aminoacyl TRNA Synthetase Complex-Interacting Multifunctional Protein 1
15	*EGLN1*	Egl-9 Family Hypoxia Inducible Factor 1
16	*C5orf22*	Chromosome 5 Open Reading Frame 22
17	*MICU2*	Mitochondrial Calcium Uptake 2
18	*NIPBL*	NIPBL, Cohesin Loading Factor
19	*N6AMT1*	N-6 adenine-specific DNA methyltransferase 1
20	*MARCH6*	Membrane Associated Ring-CH-Type Finger 6
21	*ZNF131*	Zinc Finger Protein 131
22	*SRP72*	Signal Recognition Particle 72kDa
23	*ARID5B*	AT-Rich Interaction Domain 5B
24	*CEP72*	Centrosomal Protein 72
25	*TIPRL*	TOR Signaling Pathway Regulator
26	*FAM172A*	Family With Sequence Similarity 172 Member A
27	*EMC2*	ER Membrane Protein Complex Subunit 2
28	*EDEM1*	ER Degradation Enhancing Alpha-Mannosidase Like Protein 1
29	*CYB5R4*	Cytochrome B5 Reductase 4
30	*RETN*	Resistin
31	*FUT3*	Fucosyltransferase 3 (Lewis Blood Group)

Network of genes derived from GeneMANIA based on 244 studies in humans.

Table S7: **Enriched diseases and biological functions in the gene network relevant to cardiovascular disease with p-values ranging between 1.0E-2 and 5.0E-2 (statistically less significant than the diseases and biological functions listed in Table 4) as identified by IPA based on Fisher's exact test.**

Category	Disease or Function	Genes
Cardiovascular System Development and Function	Abnormal morphology of dilated vasculature	*ANTXR2*
Lipid Metabolism	Recognition of lipid	*TLR5*
Connective Tissue Development and Function	Morphology of adipocytes	*ARID5B, RETN*
Endocrine System Development and Function	Insulin sensitivity	*CYB5R4, RETN*
Cell Signaling	Handling of Ca^{2+}	*MICU2*
Organismal Injury and Abnormalities	Infection of kidney	*TLR5*
Metabolic Disease	Susceptibility to type 2 diabetes mellitus	*RETN*
Hematological System Development and Function	Decreased hematocrit of organism	*EGLN1*
Respiratory System Development and Function	Respiratory minute volume	*EGLN1*
Cardiovascular System Development and Function	Morphogenesis of heart	*EGLN1, NIPBL*
Protein Synthesis	Quantity of hemoglobin in blood	*EGLN1*
Tissue Morphology	Morphology of connective tissue	*ARID5B, CYB5R4 RETN, TLR5*
Cellular Movement	Migration of fibroblasts	*AIMP1, ARID5B*
Cardiovascular System Development and Function	Contractility of ventricular myocytes	*TLR5*
Cellular Compromise, Hepatic System Disease	Oxidative stress response of hepatocytes	*CYB5R4*
Cell-To-Cell Signaling and Interaction	Activation of kidney cell lines	*TLR5*
Cellular Assembly and Organization, Inflammatory Response	Formation of neutrophil extracellular trap	*RETN*
Cardiovascular System Development and Function	Functional recovery of heart	*EGLN1*
Free Radical Scavenging	Metabolism of superoxide	*CYB5R4*
Endocrine System Development and Function	Quantity of insulin in blood	*CYB5R4, TLR5*
Cell Morphology, Cellular Function and Maintenance	Autophagy of cytoplasm	*TLR5*
Carbohydrate Metabolism	Synthesis of oligosaccharide	*FUT3*
Cell Death and Survival	Apoptosis of peripheral blood lymphocytes	*AIMP1*
Metabolic Disease	Hypoinsulinemia	*CYB5R4*
Cell-To-Cell Signaling and Interaction	Activation of smooth muscle cells	*RETN*
Cardiovascular Disease, Organismal Injury and Abnormalities	Dysfunction of endothelial tissue	*RETN*
Metabolic Disease	Insulin-dependent diabetes mellitus	*NRG3, RETN, TLR5*
Endocrine System Disorders, Metabolic Disease	Insulin resistance of liver	*RETN*
Protein Synthesis	Quantity of protein in blood	*CYB5R4, EGLN1, TLR5*
Cardiovascular System Development and Function	Morphogenesis of ventricular septum	*EGLN1*
Cell Morphology, Cellular Assembly and Organization	Size of mitochondria	*CYB5R4*
Connective Tissue Development and Function	Mass of adipose tissue	*CYB5R4, TLR5*
Connective Tissue Development and Function	Differentiation of fibroblast cell lines	*EGLN1, RETN*
Molecular Transport, Nucleic Acid Metabolism	Quantity of NADPH	*NNT*
Cellular Assembly and Organization	Activation of mitochondria	*RETN*
Immune Cell Trafficking, Inflammatory Response	Cellular infiltration by monocytes	*RETN*
Cellular Compromise	Stress response of cells	*CYB5R4, RETN*
Connective Tissue Development and Function	Abnormal morphology of adipocytes	*ARID5B*
Cardiovascular System Development and Function	Density of capillary vessel	*EGLN1*
Carbohydrate Metabolism	Disposal of D-glucose	*RETN*

Continued on next page

Table S7 – *Continued from previous page*

Category	Disease or Function	Genes
Hematological System Development and Function	Hematocrit of blood	*EGLN1*
Immune Cell Trafficking, Inflammatory Response	Cellular infiltration by macrophages	*EGLN1, RETN*
Cardiovascular System Development and Function	Abnormal morphology of trabeculae carne	*EGLN1*
Nutritional Disease	Obesity	*ARID5B, RETN, TLR5*
Hematological System Development and Function	Cell viability of neutrophils	*ADGRE2*
Cell-mediated Immune Response	Development of Th17 cells	*TLR5*
Hematological Disease, Immunological Disease	Growth of lymphoma	*AIMP1*
Cardiovascular System Development and Function	Morphogenesis of cardiac muscle	*EGLN1*
Connective Tissue Development and Function	Function of adipose tissue	*TLR5*
Protein Synthesis	Production of cytokine	*TLR5*
Cardiovascular System Development and Function	QT interval of heart	*NRG3*

Table S8: Interactions within the advanced coronary calcium network generated by GeneMANIA (network shown in Figure 6). The nature of interactions (INT) between gene pairs are based either on co-expression (CE) or genetic interactions (GI). We list the source of the interactions (networks previously published) under “Ref”.

#	Gene 1	Gene 2	INT	Ref	#	Gene 1	Gene 2	INT	Ref	#	Gene 1	Gene 2	INT	Ref
1	C5orf28	PAIP1	CE	[7]	48	C5orf22	C5orf28	CE	[8]	95	AIMP1	NNT	CE	[9]
2	WDR70	PAIP1	CE	[7]	49	C5orf22	NNT	CE	[8]	96	C5orf22	PAIP1	CE	[9]
3	WDR70	C5orf28	CE	[7]	50	C5orf22	WDR70	CE	[8]	97	MARCH6	NNT	CE	[9]
4	FASTKD3	PAIP1	CE	[7]	51	C5orf22	FASTKD3	CE	[8]	98	ZNF131	NNT	CE	[9]
5	FASTKD3	C5orf28	CE	[7]	52	MICU2	PAIP1	CE	[8]	99	SRP72	MICU2	CE	[9]
6	FASTKD3	WDR70	CE	[7]	53	MICU2	C5orf28	CE	[8]	100	TIPRL	TLR5	CE	[9]
7	AIMP1	C5orf28	CE	[7]	54	MICU2	AIMP1	CE	[8]	101	EMC2	AIMP1	CE	[9]
8	EGLN1	FOXN3	CE	[7]	55	NIPBL	C5orf28	CE	[8]	102	C5orf28	PAIP1	CE	[10]
9	C5orf22	NNT	CE	[7]	56	NIPBL	C5orf22	CE	[8]	103	C5orf22	NNT	CE	[10]
10	MICU2	PAIP1	CE	[7]	57	N6AMT1	C5orf28	CE	[8]	104	C5orf22	FASTKD3	CE	[10]
11	NIPBL	NNT	CE	[7]	58	N6AMT1	FASTKD3	CE	[8]	105	MICU2	FBXL17	CE	[10]
12	NIPBL	C5orf22	CE	[7]	59	MARCH6	C5orf22	CE	[8]	106	MICU2	PAIP1	CE	[10]
13	N6AMT1	C5orf28	CE	[7]	60	MARCH6	NIPBL	CE	[8]	107	ZNF131	C5orf28	CE	[10]
14	MARCH6	C5orf28	CE	[7]	61	ZNF131	WDR70	CE	[8]	108	EDEM1	ARID5B	CE	[10]
15	MARCH6	FASTKD3	CE	[7]	62	ZNF131	FASTKD3	CE	[8]	109	FBXL17	ANTXR2	GI	[11]
16	MARCH6	C5orf22	CE	[7]	63	ZNF131	C5orf22	CE	[8]	110	YAE1D1	ANTXR2	GI	[11]
17	MARCH6	NIPBL	CE	[7]	64	ARID5B	FOXN3	CE	[8]	111	PAIP1	YAE1D1	GI	[11]
18	ZNF131	PAIP1	CE	[7]	65	CEP72	FASTKD3	CE	[8]	112	FOXN3	FBXL17	GI	[11]
19	ZNF131	WDR70	CE	[7]	66	FAM172A	C5orf28	CE	[8]	113	NNT	ANTXR2	GI	[11]
20	ZNF131	FASTKD3	CE	[7]	67	FAM172A	MARCH6	CE	[8]	114	NNT	FOXN3	GI	[11]
21	ZNF131	C5orf22	CE	[7]	68	EMC2	PAIP1	CE	[8]	115	WDR70	EMR2	GI	[11]
22	ZNF131	MICU2	CE	[7]	69	EDEM1	NNT	CE	[8]	116	AIMP1	MACC1	GI	[11]
23	ZNF131	NIPBL	CE	[7]	70	C5orf28	YAE1D1	CE	[12]	117	AIMP1	YAE1D1	GI	[11]
24	ZNF131	MARCH6	CE	[7]	71	FASTKD3	YAE1D1	CE	[12]	118	AIMP1	PAIP1	GI	[11]
25	SRP72	C5orf28	CE	[7]	72	AIMP1	YAE1D1	CE	[12]	119	EGLN1	FOXN3	GI	[11]
26	SRP72	AIMP1	CE	[7]	73	AIMP1	C5orf28	CE	[12]	120	C5orf22	FOXN3	GI	[11]
27	ARID5B	FOXN3	CE	[7]	74	MICU2	WDR70	CE	[12]	121	MICU2	ANTXR2	GI	[11]
28	CEP72	PAIP1	CE	[7]	75	TIPRL	SRP72	CE	[12]	122	MICU2	FOXN3	GI	[11]
29	CEP72	C5orf28	CE	[7]	76	FAM172A	WDR70	CE	[12]	123	NIPBL	ANTXR2	GI	[11]
30	TIPRL	C5orf28	CE	[7]	77	EMC2	YAE1D1	CE	[12]	124	NIPBL	EMR2	GI	[11]
31	TIPRL	FASTKD3	CE	[7]	78	EMC2	C5orf28	CE	[12]	125	N6AMT1	FBXL17	GI	[11]
32	TIPRL	ZNF131	CE	[7]	79	EMC2	FASTKD3	CE	[12]	126	N6AMT1	FOXN3	GI	[11]
33	FAM172A	FOXN3	CE	[7]	80	CYB5R4	MICU2	CE	[12]	127	MARCH6	FBXL17	GI	[11]
34	EMC2	C5orf28	CE	[7]	81	RETN	EMR2	CE	[12]	128	MARCH6	YAE1D1	GI	[11]
35	EMC2	AIMP1	CE	[7]	82	FUT3	MACC1	CE	[12]	129	SRP72	PAIP1	GI	[11]
36	EMC2	SRP72	CE	[7]	83	NNT	PAIP1	CE	[13]	130	SRP72	FOXN3	GI	[11]
37	EDEM1	EMR2	CE	[7]	84	NIPBL	FOXN3	CE	[13]	131	SRP72	NIPBL	GI	[11]
38	CYB5R4	PAIP1	CE	[7]	85	NIPBL	NNT	CE	[13]	132	FAM172A	MICU2	GI	[11]
39	RETN	TLR5	CE	[7]	86	MARCH6	PAIP1	CE	[13]	133	EMC2	YAE1D1	GI	[11]
40	FUT3	TLR5	CE	[7]	87	MARCH6	NIPBL	CE	[13]	134	EMC2	AIMP1	GI	[11]
41	C5orf28	PAIP1	CE	[8]	88	SRP72	FOXN3	CE	[13]	135	EDEM1	FBXL17	GI	[11]
42	WDR70	C5orf28	CE	[8]	89	TIPRL	NNT	CE	[13]	136	EDEM1	C5orf28	GI	[11]
43	WDR70	NNT	CE	[8]	90	FAM172A	NNT	CE	[13]	137	EDEM1	MARCH6	GI	[11]
44	FASTKD3	PAIP1	CE	[8]	91	FAM172A	NIPBL	CE	[13]	138	CYB5R4	FBXL17	GI	[11]
45	FASTKD3	C5orf28	CE	[8]	92	FAM172A	MARCH6	CE	[13]	139	CYB5R4	FOXN3	GI	[11]
46	EGLN1	NNT	CE	[8]	93	NNT	PAIP1	CE	[9]	140	CYB5R4	NNT	GI	[11]
47	C5orf22	PAIP1	CE	[8]	94	AIMP1	PAIP1	CE	[9]	141	RETN	C5orf28	GI	[11]
										142	FUT3	AIMP1	GI	[11]

References

1. Ferguson, J.F., Matthews, G.J., Townsend, R.R., Raj, D.S., Kanetsky, P.A., Budoff, M., Fischer, M.J., Rosas, S.E., Kanthety, R., Rahman, M., *et al.*: Candidate gene association study of coronary artery calcification in chronic kidney disease: findings from the CRIC study (Chronic Renal Insufficiency Cohort). Journal of the American College of Cardiology **62**(9), 789–798 (2013)

2. Wojczynski, M.K., Li, M., Bielak, L.F., Kerr, K.F., Reiner, A.P., Wong, N.D., Yanek, L.R., Qu, L., White, C.C., Lange, L.A., *et al.*: Genetics of coronary artery calcification among African Americans, a meta-analysis. BMC medical genetics **14**(1), 75 (2013)

3. van Setten, J., Isgum, I., Smolonska, J., Ripke, S., de Jong, P.A., Oudkerk, M., de Koning, H., Lammers, J.-W.J., Zanen, P., Groen, H.J., *et al.*: Genome-wide association study of coronary and aortic calcification implicates risk loci for coronary artery disease and myocardial infarction. Atherosclerosis **228**(2), 400–405 (2013)

4. O'Donnell, C.J., Kavousi, M., Smith, A.V., Kardia, S.L., Feitosa, M.F., Hwang, S.-J., Sun, Y.V., Province, M.A., Aspelund, T., Dehghan, A., *et al.*: Genome-wide association study for coronary artery calcification with follow-up in myocardial infarction. Circulation **124**(25), 2855–2864 (2011)

5. O'Donnell, C.J., Cupples, L.A., D'Agostino, R.B., Fox, C.S., Hoffmann, U., Hwang, S.-J., Ingellson, E., Liu, C., Murabito, J.M., Polak, J.F., *et al.*: Genome-wide association study for subclinical atherosclerosis in major arterial territories in the NHLBI's Framingham Heart Study. BMC medical genetics **8**(Suppl 1), 4 (2007)

6. Polfus, L.M., Smith, J.A., Shimmin, L.C., Bielak, L.F., Morrison, A.C., Kardia, S.L., Peyser, P.A., Hixson, J.E.: Genome-wide association study of gene by smoking interactions in coronary artery calcification. PloS one **8**(10), 74642 (2013)

7. Gysin, S., Paquette, J., McMahon, M.: Analysis of mrna profiles after mek1/2 inhibition in human pancreatic cancer cell lines reveals pathways involved in drug sensitivity. Molecular Cancer Research **10**(12), 1607–1619 (2012)

8. Salaverria, I., Philipp, C., Oschlies, I., Kohler, C.W., Kreuz, M., Szczepanowski, M., Burkhardt, B., Trautmann, H., Gesk, S., Andrusiewicz, M., *et al.*: Translocations activating irf4 identify a subtype

of germinal center-derived b-cell lymphoma affecting predominantly children and young adults. Blood **118**(1), 139–147 (2011)

9. Bild, A.H., Yao, G., Chang, J.T., Wang, Q., Potti, A., Chasse, D., Joshi, M.-B., Harpole, D., Lancaster, J.M., Berchuck, A., *et al.*: Oncogenic pathway signatures in human cancers as a guide to targeted therapies. Nature **439**(7074), 353–357 (2006)

10. Bahr, T.M., Hughes, G.J., Armstrong, M., Reisdorph, R., Coldren, C.D., Edwards, M.G., Schnell, C., Kedl, R., LaFlamme, D.J., Reisdorph, N., *et al.*: Peripheral blood mononuclear cell gene expression in chronic obstructive pulmonary disease. American journal of respiratory cell and molecular biology **49**(2), 316–323 (2013)

11. Lin, A., Wang, R.T., Ahn, S., Park, C.C., Smith, D.J.: A genome-wide map of human genetic interactions inferred from radiation hybrid genotypes. Genome research **20**(8), 1122–1132 (2010)

12. Innocenti, F., Cooper, G.M., Stanaway, I.B., Gamazon, E.R., Smith, J.D., Mirkov, S., Ramirez, J., Liu, W., Lin, Y.S., Moloney, C., *et al.*: Identification, replication, and functional fine-mapping of expression quantitative trait loci in primary human liver tissue. PLoS Genet **7**(5), 1002078 (2011)

13. Wang, Q., Diskin, S., Rappaport, E., Attiyeh, E., Mosse, Y., Shue, D., Seiser, E., Jagannathan, J., Shusterman, S., Bansal, M., *et al.*: Integrative genomics identifies distinct molecular classes of neuroblastoma and shows that multiple genes are targeted by regional alterations in dna copy number. Cancer research **66**(12), 6050–6062 (2006)
