# Supplementary material for: Genotype-driven identification of a molecular network predictive of advanced coronary calcium in ClinSeq® and Framingham Heart Study cohorts
Source: BMC Syst Biol. 2017 Oct 26;11:99. doi: 10.1186/s12918-017-0474-5 (PMC5659034; doi:10.1186/s12918-017-0474-5)
Supplement: Supplementary file 2 — This pdf file provides information about the genotype data from ClinSeq®; and FHS cohorts, rationale behind random forest and neural network implementation for modeling advanced CAC, and mouse and rat model-based associations between the predictive network genes and cardiovascular disease processes and risk factors. (PDF 165 kb) [file 12918_2017_474_MOESM2_ESM.pdf]

Supplementary Text

Details regarding the genotype data from ClinSeq® and FHS cohorts

For the ClinSeq® cohort [1], SNP genotyping was performed using HumanOmni2.5 Illumina BeadChip arrays. Genotyping was carried out with in accordance with the Illumina Infinium assay protocol. In brief, this involved amplification of DNA by whole genome amplification (WGA), hybridization of the WGA product to the BeadArray (an array-based enzymatic reaction extending captured SNP targets by incorporating biotin-labeled dNTP nucleotides into appropriate allele specific probe), and detection and signal amplification to read the incorporated labels. The BeadChips were scanned using the Illumina iScan system and processed with the GenomeStudio v2011.1 Genotyping module. The BeadChips consist of specific 50-mer oligonucleotide probe arrays at an average of 30-fold redundancy. The design of the HumanOmni2.5 BeadChips incorporates around 2.5 million markers. GenomeStudio output files were processed using a custom Perl script to derive the nucleotides at each SNP position for each subject.

For the FHS cohort, genotyping data was compiled from three resources. More than 276,000 variants from the Illumina Infinium Human Exome Array v1.0 was genotyped and jointly called as part of the Cohorts for Heart and Aging Research in Genomic Epidemiology (CHARGE) Consortium [2]. The Framingham SNP Health Association Resource (SHARe) project [3] used the Affymetrix 500K mapping array and the Affymetrix 50K supplemental gene focused array resulted in 503,551 SNPs with successful call rate >95% and Hardy-Weinberg equilibrium (HWE) P>1.0E-6 (based on an exact test [4, 5] that quantifies the deviation from HWE). Additional genotype imputation was conducted based on this SHARe data using Minimac with reference panel from the 1000 Genomes Project (Version Phase 1 integrated release v3, April 2012, all population). Best-guessed genotypes were used for markers that were not available from the first two actual genotyping platforms.

The total number of SNPs in our study was 113 (combination of SNP sets 1 and 2). All of these SNPs were identified from the ClinSeq discovery cohort (based on HumanOmni2.5 Illumina BeadChip arrays, no imputation). Since we needed the genotypes of these 113 SNPs for all cases and controls in the FHS replication cohort, we used a liberal imputation quality threshold of 0.3 (79 of 113 SNPs imputed). Only two of the 79 SNPs had imputation qualities less than 0.49, and neither of these two SNPs had positive predictive power in RF models of the FHS cohort. Furthermore, out of the 21 SNPs that general optimal predictive performance in both cohorts (Table 3), 16 were imputed. Among these 16 SNPs, imputation quality ranged between 0.82-0.99 (median: 0.99 and interquartile range: 0.97-0.99). Therefore, the lenient imputation quality threshold of 0.3 did not have an impact on our results and conclusions, while allowing

us to test whether we can replicate the predictive patterns from the discovery cohort within the replication cohort.

Rationale behind random forest and neural network implementation for modeling advanced CAC

The basic random forest model implementation assigns a predictive importance value to each predictor (as described in the "Methods" section), a feature that lacks in the basic neural network implementation. We used these predictive importance values to rank predictors and eliminated features with negative predictive importance and. Because, such features reduced the overall predictive performance. In both cohorts, focusing on features with positive predictive performance allowed us to compare the predictive performance over a range of predictors (e.g. top 5-20 predictors) without having to worry about features that may significantly reduce the overall predictive performance. As a result, we were able to check for the consistency of major predictive patterns in both cohorts (e.g. predictive power of SNP Set-2 with and without clinical variables).

By utilizing the random forest based predictor ranking, we also gradually reduced the number of features and identified optimal sets of features. 21 SNPs in SNP Set-2 were optimal since they generated the best predictive performance in terms of areas under receiver operating characteristics curves (ROC-AUC). After identifying these 21 SNPs as optimal set of predictors, the basic neural network implementation was chosen for training models with one group of patients (discovery cohort) and testing with another group (replication cohort) for the following reasons:

(1) Neural networks, which rely on sums of weighted inputs transformed by transfer functions to generate model outputs, operate very differently than the decision tree based random forests that don't utilize transfer functions. This difference allows us to test whether the cumulative predictive signal captured by random forests is also captured by a completely different method, a good way to test for the robustness of the predictive signals in our data.

(2) Neural networks rely on linear and nonlinear transfer functions and complex topologies for modeling predictor-predictor and predictor-output relationships. We were able to test several neural network topologies and check for the robustness of predictive power independent of a single network topology, whereas we had significantly less ability to sample over random forest topologies in the same manner, since random forest topologies are automatically shaped by the random forest algorithm based on training data.

(3) It is not possible to use out-of- bag sampling both to train random forests with one cohort and to test with another cohort, unless we merge the two cohorts prior to modeling. However, this would defeat the purpose of having independent discovery and replication cohorts. If we were to test random forests

(discovery cohort trained) with all replication cohort samples (not out-of- bag sampling), the subtle training data-specific topological features of individual trees would lead to poor predictive performance. On the other hand, neural networks provide full control over training and testing samples (unlike the randomized out-of-sampling), which enables training with discovery cohort data and testing with replication cohort data. Hence, each neural network topology allows integration of discovery and replication cohort data into a single model, whereas we have separate random forest models for the two cohorts.

To recap, we used random forest models for feature ranking and selection. Then, based on the selection of best set of features, we trained neural networks with discovery cohort (ClinSeq) data and subsequently tested them with replication cohort (FHS) data. Within neural networks, we used two hidden layers to look for potential linear and nonlinear interactions between model inputs. Using two hidden layers helped us achieve this goal in a more comprehensive way in comparison to using a single hidden layer. Since we have 21 inputs (SNPs from SNP Set-2) for NN models, we include 1-20 nodes per hidden layer to make the maximum number of nodes approximately equal to the number of inputs. This way we avoided overly complex neural network topologies. In addition, training overly complex neural network topologies (>20 nodes per hidden layer) with the data from discovery cohort can easily lead to low predictive performance when the same networks are tested with data from the independent replication cohort. Hence, this choice serves as a precaution against overfitting with training data.

In summary, using random forests first and neural networks second allowed us to utilize the complementary features of these two machine learning methods. Furthermore, using one machine learning method for feature selection and feeding the selected features (e.g., optimal set) into another method (rather than relying on a single method throughout a study) is considered as one of the best practices in the machine learning literature [6].

Associations between predictive network genes and cardiovascular disease processes and risk factors identified through mouse and rat models

Several mouse models have linked *ARID5B* (a transcription factor involved in smooth muscle cell differentiation and proliferation) to obesity, differentiation of adipocytes, amount of white and brown adipose tissue, percentage body fat, and abnormal morphology of fat cells [7–11]. Similarly, multiple mouse models [8, 12–14] showed that *CYB5R4* (involved in endoplasmic reticulum (ER) stress response pathway and glucose homeostasis) is associated with mass of adipose tissue, hypoinsulinemia, hyperglycemia, secretion of insulin, rate of oxidation of fatty acid, hyperlipidemia, timing of the onset of hyperglycemia, and diabetes.

Similarly, using mouse model-based studies, *EGLN1* (involved in the regulation of angiogenesis, oxygen homeostasis, and response to nitric oxide) and its paralog *EGLN3* have been linked to the necrosis of heart tissue, apoptosis of cardiomyocytes in infarcted mouse heart, stabilization of HIF1-alpha protein (associated with atherosclerotic plaques [15]) in left ventricle from mouse heart, functional recovery of heart, hepatic steatosis (fatty liver disease), angiectasis (abnormal dilation of blood vessels), and dilated cardiomyopathy (reduced ability of heart to pump blood due to enlarged and weakened left ventricle) [16–20]. Through mouse and rat models, *RETN* (a biomarker for metabolic syndrome, atherosclerosis, and insulin-dependent diabetes, and a regulator of collagen metabolic process and smooth muscle cell migration) has been linked to insulin resistance, hyperinsulinemia, glucose intolerance, quantity of D-Glucose, quantity of circulating free fatty acid, LDLR, reactive oxygen species, and triglycerides [21–26], as well as increased atherosclerotic progression [27]. Several rat and mouse models showed that *TLR5* (a transmembrane receptor involved in inflammatory response, nitric oxide biosynthesis, and cellular response to lipopolysaccharide) is associated with obesity, hypertension, insulin resistance, autoimmune diabetes, cholesterol and triglyceride levels, systolic and diastolic blood pressure in systemic artery, and inflammation [28–30]. Finally, *NRG3* serves as a ligand of the tyrosine kinase receptor ErbB4 that has been shown to affect the development of heart and the flow of blood in heart in multiple mouse models [31–34].

References

1. **The ClinSeq Project: Piloting large-scale genome sequencing for research in genomic medicine** 2016, [https://www.ncbi.nlm.nih.gov/projects/gap/cgi-bin/study.cgi?study_id=phs000971.v1.p1].
2. **CHARGE (Cohorts for Heart and Aging Research in Genomic Epidemiology) Consortium Summary Results from Genomic Studies** 2016, [https://www.ncbi.nlm.nih.gov/projects/gap/cgi-bin/study.cgi?study_id=phs000930.v4.p1].
3. **Framingham SNP Health Association Resource (SHARe) project** 2016, [http://www.ncbi.nlm.nih.gov/projects/gap/cgi-bin/study.cgi?study_id=phs000007.v10.p5].
4. Wigginton JE, Cutler DJ, Abecasis GR: **A note on exact tests of Hardy-Weinberg equilibrium**. *The American Journal of Human Genetics* 2005, **76**(5):887–893.
5. Purcell S, Neale B, Todd-Brown K, Thomas L, Ferreira MA, Bender D, Maller J, Sklar P, De Bakker PI, Daly MJ, et al.: **PLINK: a tool set for whole-genome association and population-based linkage analyses**. *The American Journal of Human Genetics* 2007, **81**(3):559–575.
6. Malley JD, Malley KG, Pajevic S: *Statistical learning for biomedical data*. Cambridge University Press 2011.
7. Whitson RH, Tsark W, Huang TH, Itakura K: **Neonatal mortality and leanness in mice lacking the ARID transcription factor Mrf-2**. *Biochemical and biophysical research communications* 2003, **312**(4):997–1004.
8. Rankinen T, Zuberi A, Chagnon YC, Weisnagel SJ, Argyropoulos G, Walts B, Pérusse L, Bouchard C: **The human obesity gene map: the 2005 update**. *Obesity* 2006, **14**(4):529–644.
9. Yamakawa T, Whitson RH, Li SL, Itakura K: **Modulator recognition factor-2 is required for adipogenesis in mouse embryo fibroblasts and 3T3-L1 cells**. *Molecular Endocrinology* 2008, **22**(2):441–453.

10. Lahoud MH, Ristevski S, Venter DJ, Jermiin LS, Bertoncello I, Zavarsek S, Hasthorpe S, Drago J, de Kretser D, Hertzog PJ, et al.: **Gene targeting of Desrt, a novel ARID class DNA-binding protein, causes growth retardation and abnormal development of reproductive organs**. *Genome research* 2001, **11**(8):1327–1334.

11. Hata K, Takashima R, Amano K, Ono K, Nakanishi M, Yoshida M, Wakabayashi M, Matsuda A, Maeda Y, Suzuki Y, et al.: **Arid5b facilitates chondrogenesis by recruiting the histone demethylase Phf2 to Sox9-regulated genes**. *Nature communications* 2013, **4**.

12. Xie J, Zhu H, Larade K, Ladoux A, Seguritan A, Chu M, Ito S, Bronson RT, Leiter EH, Zhang CY, et al.: **Absence of a reductase, NCB5OR, causes insulin-deficient diabetes**. *Proceedings of the National Academy of Sciences of the United States of America* 2004, **101**(29):10750–10755.

13. Xu M, Wang W, Frontera JR, Neely MC, Lu J, Aires D, Hsu FF, Turk J, Swerdlow RH, Carlson SE, et al.: **Ncb5or deficiency increases fatty acid catabolism and oxidative stress**. *Journal of Biological Chemistry* 2011, **286**(13):11141–11154.

14. Zhang Y, Larade K, Jiang Zg, Ito S, Wang W, Zhu H, Bunn HF: **The flavoheme reductase Ncb5or protects cells against endoplasmic reticulum stress-induced lipotoxicity**. *Journal of lipid research* 2010, **51**:53–62.

15. Rahtu-Korpela L, Määttä J, Dimova EY, Hörkkö S, Gylling H, Walkinshaw G, Hakkola J, Kivirikko KI, Myllyharju J, Serpi R, et al.: **Hypoxia-inducible factor-prolyl 4-hydroxylase-2 inhibition protects against development of atherosclerosis**. *Arteriosclerosis, thrombosis, and vascular biology* 2016, :ATVBAHA–115.

16. Hölscher M, Silter M, Krull S, von Ahlen M, Hesse A, Schwartz P, Wielockx B, Breier G, Katschinski DM, Zieseniss A: **Cardiomyocyte-specific prolyl-4-hydroxylase domain 2 knock out protects from acute myocardial ischemic injury**. *Journal of Biological Chemistry* 2011, **286**(13):11185–11194.

17. Eckle T, Köhler D, Lehmann R, El Kasmi KC, Eltzschig HK: **Hypoxia-inducible factor-1 is central to cardioprotection a new paradigm for ischemic preconditioning**. *Circulation* 2008, **118**(2):166–175.

18. Takeda K, Ho VC, Takeda H, Duan LJ, Nagy A, Fong GH: **Placental but not heart defects are associated with elevated hypoxia-inducible factor α levels in mice lacking prolyl hydroxylase domain protein 2**. *Molecular and cellular biology* 2006, **26**(22):8336–8346.

19. Minamishima YA, Moslehi J, Padera RF, Bronson RT, Liao R, Kaelin WG: **A feedback loop involving the Phd3 prolyl hydroxylase tunes the mammalian hypoxic response in vivo**. *Molecular and cellular biology* 2009, **29**(21):5729–5741.

20. Takeda K, Cowan A, Fong GH: **Essential role for prolyl hydroxylase domain protein 2 in oxygen homeostasis of the adult vascular system**. *Circulation* 2007, **116**(7):774–781.

21. Satoh H, Nguyen MA, Miles PD, Imamura T, Usui I, Olefsky JM: **Adenovirus-mediated chronic hyper-resistinemia leads to in vivo insulin resistance in normal rats**. *The Journal of clinical investigation* 2004, **114**(2):224–231.

22. Rajala MW, Obici S, Scherer PE, Rossetti L: **Adipose-derived resistin and gut-derived resistin-like molecule–β selectively impair insulin action on glucose production**. *The Journal of clinical investigation* 2003, **111**(2):225–230.

23. Steppan CM, Bailey ST, Bhat S, Brown EJ, Banerjee RR, Wright CM, Patel HR, Ahima RS, Lazar MA: **The hormone resistin links obesity to diabetes**. *Nature* 2001, **409**(6818):307–312.

24. Sato N, Kobayashi K, Inoguchi T, Sonoda N, Imamura M, Sekiguchi N, Nakashima N, Nawata H: **Adenovirus-mediated high expression of resistin causes dyslipidemia in mice**. *Endocrinology* 2005, **146**:273–279.

25. Pravenec M, Kazdová L, Landa V, Zídek V, Mlejnek P, Jansa P, Wang J, Qi N, Kurtz TW: **Transgenic and recombinant resistin impair skeletal muscle glucose metabolism in the spontaneously hypertensive rat**. *Journal of Biological Chemistry* 2003, **278**(46):45209–45215.

26. Kim KH, Zhao L, Moon Y, Kang C, Sul HS: **Dominant inhibitory adipocyte-specific secretory factor (ADSF)/resistin enhances adipogenesis and improves insulin sensitivity**. *Proceedings of the National Academy of Sciences of the United States of America* 2004, **101**(17):6780–6785.

27. Asterholm IW, Rutkowski JM, Fujikawa T, Cho YR, Fukuda M, Tao C, Wang ZV, Gupta RK, Elmquist JK, Scherer PE: **Elevated resistin levels induce central leptin resistance and increased atherosclerotic progression in mice**. *Diabetologia* 2014, **57**(6):1209–1218.

28. Vijay-Kumar M, Aitken JD, Carvalho FA, Cullender TC, Mwangi S, Srinivasan S, Sitaraman SV, Knight R, Ley RE, Gewirtz AT: **Metabolic syndrome and altered gut microbiota in mice lacking Toll-like receptor 5**. *Science* 2010, **328**(5975):228–231.

29. Guo LH, Guo KT, Wendel HP, Schluesener HJ: **Combinations of TLR and NOD2 ligands stimulate rat microglial P2X4R expression**. *Biochemical and biophysical research communications* 2006, **349**(3):1156–1162.

30. Feuillet V, Medjane S, Mondor I, Demaria O, Pagni PP, Galán JE, Flavell RA, Alexopoulou L: **Involvement of Toll-like receptor 5 in the recognition of flagellated bacteria**. *Proceedings of the National Academy of Sciences* 2006, **103**(33):12487–12492.

31. Elenius K, Paatero I: **ErbB4 and its isoforms: patentable drug targets?** *Recent patents on DNA & gene sequences* 2008, **2**:27–33.

32. Carpenter G: **ErbB-4: mechanism of action and biology**. *Experimental cell research* 2003, **284**:66–77.

33. Yarden Y, Sliwkowski MX: **Untangling the ErbB signalling network**. *Nature reviews Molecular cell biology* 2001, **2**(2):127–137.

34. Tidcombe H, Jackson-Fisher A, Mathers K, Stern DF, Gassmann M, Golding JP: **Neural and mammary gland defects in ErbB4 knockout mice genetically rescued from embryonic lethality**. *Proceedings of the National Academy of Sciences* 2003, **100**(14):8281–8286.
